# Anaplastic Lymphoma Kinase (ALK)-Negative Inflammatory Myofibroblastic Tumor of the Kidney in a Nine-Month-Old Girl

**DOI:** 10.7759/cureus.23289

**Published:** 2022-03-18

**Authors:** Bisma Tareen, Hayden Faith, Abdul Waheed, Asad Ullah, Sravan K Kavuri

**Affiliations:** 1 Internal Medicine, Bolan Medical College, Quetta, PAK; 2 Oncology, Medical College of Georgia-Augusta University, Augusta, USA; 3 Surgery, San Joaquin General Hospital, San Joaquin, USA; 4 Pathology, Medical College of Georgia-Augusta University, Augusta, USA

**Keywords:** hematuria, ct (computed tomography) imaging, wilms tumor, inflammatory myofibroblastic tumor, benign

## Abstract

An inflammatory myofibroblastic tumor (IMT) is an uncommon, benign tumor of myofibroblastic spindle cells. An IMT can occur in any part of the body. However, involving kidney is exceedingly rare. When this rare entity occurs in children, it becomes incredibly challenging to distinguish this rare entity from other malignancies such as Wilms tumor. Although imaging studies of the abdomen and pelvis add to the diagnosis, however, histological examination and immunohistochemical staining remain the gold standard for the precise diagnosis of this rare entity. To the best of our knowledge, only 48 cases of renal IMT have been published in the medical literature so far. We report the case of a nine-month-old girl who was brought with complaints of hematuria, and later, imaging and histological confirmation revealed an anaplastic lymphoma kinase (ALK)-negative IMT of the kidney.

## Introduction

An inflammatory myofibroblastic tumor (IMT) is a rare benign lesion [1]. The various terms used are plasma cell granuloma, inflammatory pseudotumor, fibroxanthoma, and xanthogranuloma [1]. It can affect any organ/system of the body, including lungs, gastrointestinal tract (mesentery), omentum, liver, head and neck, and urogenital system, with lungs being the most common site of occurrence [2,3]. Renal IMT, however, is very rare, and only a limited number of cases have been reported in the medical literature [2]. IMT can occur in any age group; however, it usually occurs in children and young adults [1,2].

In addition, the pathogenesis of this atypical disease is unknown and worth discussing among the medical community. However, preliminary research suggests that an inflammatory response following surgery, trauma, or infection may contribute to the pathogenesis of IMT [4,5]. Recent data emphasize the neoplastic component of the disease and suggest it as a possible cause due to its local aggressive behavior and evidence of cytogenetic clonality with overexpression of the anaplastic lymphoma kinase (ALK) gene [5,6]. Also, the clinical manifestations of the IMT are mainly site specific, and, in the case of renal IMT, the patient usually presents with hematuria lumbar pain [7]. Moreover, it rarely presents constitutional signs and symptoms, including fever, weight loss, and growth retardation [8].

Moreover, a CT or MRI of the abdomen or pelvis can help diagnose, but they are not specific; thus, histological confirmation is required [9]. The treatment also differs depending on the location and severity of the tumor [9]. Some patients benefit from long-term steroid therapy, while others require nephrectomy. We present a case of the nine-month-old girl with hematuria, proved IMT histologically after a nephrectomy.

## Case presentation

A nine-month-old female with no significant medical history was brought by parents who noticed two episodes of gross hematuria. She has a family history of breast cancer in her maternal aunt; however, there was no family history of childhood cancer or kidney disease. Gross blood was noted in the diaper on physical examination, but the examination was otherwise unremarkable. Laboratory data evaluation showed a leukocytosis of 15,700/mm^3^, RBC of 4.23 million/mm^3^, hemoglobin of 10.9 g/dL, hematocrit of 32.1%, mean corpuscular volume of 75.8 fL (low), mean corpuscular hemoglobin concentration of 34.1 g/dL, red cell distribution width of 12.6%, and thrombocytosis of 507,000/mm^3^. Urine analysis (UA) was notable for large blood with too numerous to count (TNTC) RBC, protein 30 mg/dL, 10-20 WBC/per high powerfield (PHF), trace bacteria, and negative leukocyte and nitrite.

**Figure 1:**
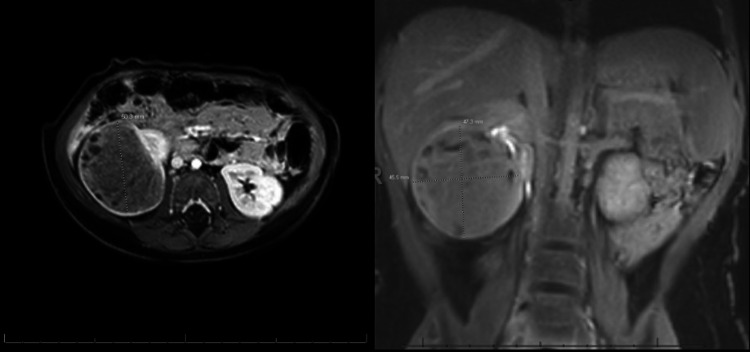
MRI revealing well-defined, predominantly solid hypoenhancing right renal mass with a small cystic area in the periphery of the lesion

On basal metabolic profile (BMP), the blood urea nitrogen (BUN) was 5 mg/dL (low), creatinine was 0.2 mg/dL (low), and calcium was 10.8 mg/dL (high). All other electrolytes were within normal limits. Additionally, the retroperitoneal ultrasonography revealed hypoechoic mass within the lower pole of the right kidney. On imaging, the MRI of the abdomen and pelvis demonstrated a well-defined, predominantly solid hypoenhancing mass involving the inferior lateral aspect of the right kidney measuring 4.7 x 4.5 x 5 cm with small cystic areas in the lesion's periphery (Figure 1).

**Figure 2:**
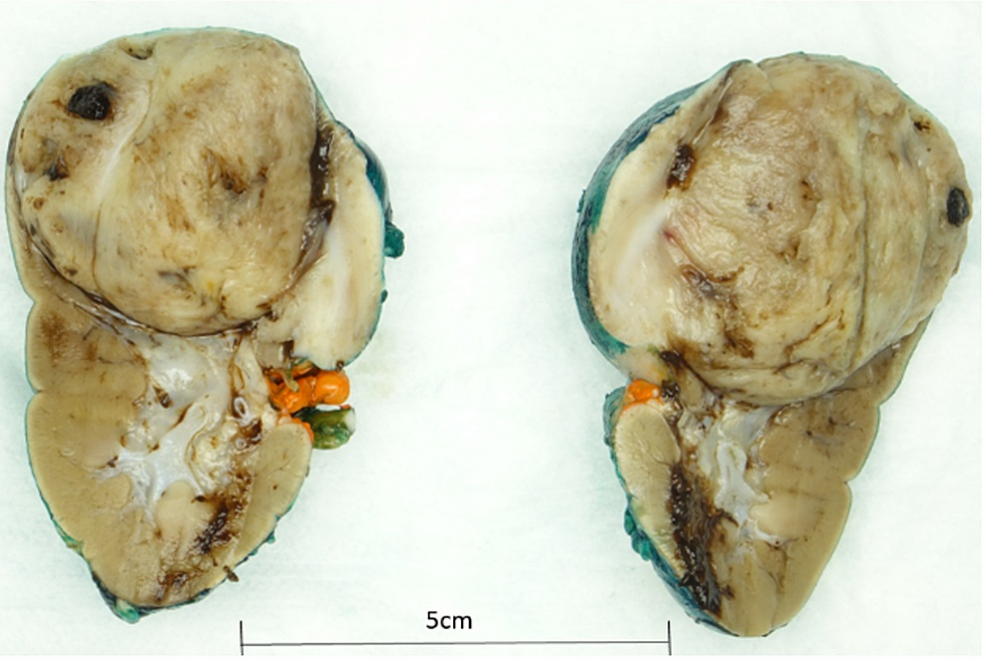
Gross examination: well-circumscribed lesion with focal hemorrhage

Based on age, presentation, and appearance on ultrasound (US) and MRI, a diagnosis of Wilms tumor/mesoblastic nephroma was considered, and a surgical consult for resection was made. Right open nephrectomy and excision of two paraaortic, two paracaval, and one hilar lymph node were conducted; the operative course was uneventful with minimal bleeding. Gross examination revealed encapsulated lesion with a tan-white cut surface, focal areas of hemorrhage, and no identifiable necrosis (Figure 2).

**Figure 3:**
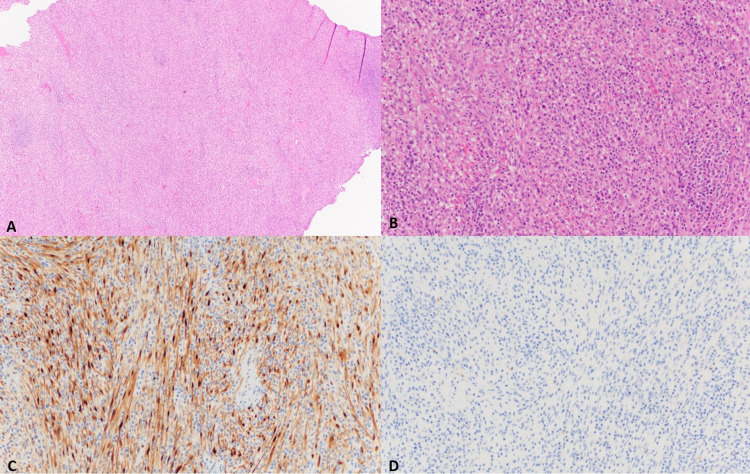
Histological examination: hematoxylin and eosin stain (H&E) A, B, H&E and bland spindle cells with mixed inflammatory infiltrate. C, Spindle cells showed nuclear and cytoplasmic positivity for cyclin D1. D, Spindle cells are negative for ALK stain. ALK: anaplastic lymphoma kinase.

Histopathological examination revealed bland spindled cells, arranged in lobules, and separated by aggregates of lymphocytes and plasma cells consistent with IMT. Immunohistochemical staining revealed that the tumor cells were positive for cyclin D1 positivity and negative for desmin, smooth muscle actin (SMA), pankeratin, epithelial membrane antigen (EMA), calponin, ALK-1, Wilms tumor (WT)-1, S100, and antimelanoma monoclonal antibody (HMB45) (Figure 3).

The lesion was completely excised with negative margins, and the tumor's regional lymph nodes were negative. The patient stayed at the hospital for a total of eight days without complications. The patient was discharged with follow-up with hematology/oncology and pediatric surgery two days later. The patient was discussed in the pediatric multidisciplinary tumor board, and follow-up with imaging was recommended. Positron emission tomography (PET)/CT scan on two-month follow-up showed no evidence of distant metastasis. The patient will have disease monitoring with magnetic resonance imaging and chest x-ray every three months for the first two to three years after resection, followed by abdomen ultrasound/chest x-ray.

## Discussion

IMT is a rare benign tumor and affects people of all ages and genders but mainly younger people [1]. Trauma, surgery, calculous pyelonephritis, and an immunological disorder like systemic lupus are possible differentials for IMT [7]. For patients with renal IMT, the most common signs and symptoms are abdominal pain, hematuria, fever, and recurrent urinary tract infections [4]. The World Health Organization (WHO) classifies IMT as a benign mesenchymal tumor with cancerous potential and divides IMT into adult and pediatric tumors [5]. According to the most recent data, these renal mesenchymal tumors are various disorders with variable histological characteristics [10].

However, no single factor has been found to be responsible for the development of this unusual condition [8]. The IMT, however, can be triggered by a variety of illnesses, including viral or autoimmune disorders. Some pathogenic organisms, like Actinomyces, Pseudomonas species, and mycoplasmal infections, have been related to IMT of the kidneys based on the scarcely published data on this condition [2]. In some cases of IMT, a positive test for Epstein-Barr virus latent membrane protein has been identified, increasing the likelihood that this virus is the causal culprit. Additionally, several IMTs have been associated with immunoglobulin G4 (IgG4) deficiencies. In this systemic and multifunctional disease, the presence of plasma and T lymphocytes expressing the IgG4 antigen is a characteristic. The presence of IgG4-related renal IMT can also be detected by measuring serum IgG4 levels [11].

Also, with the advances of medical techniques in the recent era, the diagnosis of IMT can be made using various immunological stains [12]. Based on immunohistochemical analysis, it is discovered that the proteins, vimentin, muscle-specific actin (MSA), smooth muscle actin (SMA), and creatine kinase (CK), were all detectable in IMT [12]. Furthermore, according to the findings of Li et al., the positive rates for vimentin, SMA, desmin, ALK, and CK in 30 patients with extrapulmonary IMT were 100%, 70%, 27%, 27%, and 13%, respectively [13].

The diagnosis of the IMT is challenging, and imaging using CT and MRI of the abdomen and pelvis with or without contrast alone is insufficient for the precise diagnosis [2]. The radiographic findings in renal IMT are not well understood. Furthermore, there are no published studies or guidelines pertaining to the most appropriate imaging approach for diagnosis and follow-up. With MRI, the lesion indicates low signal intensity on T1-weighted images but high signal intensity on T2-weighted images [12].

Because the imaging is unrevealing for the confirmation of the disease, histological confirmation is the gold standard for diagnosing IMT [8,10,12]. A proliferation of spindle cells admixed with varying degrees of lymphoplasmacytic infiltration characterizes IMT histologically [8,10]. The ability to identify IMT from its histological mimics, which can include both reactive processes and potentially malignant tumors, is critical in ensuring effective patient care. Lovly et al. have characterized several histological patterns of IMT, including the myxoid-vascular pattern, the compact spindle cell pattern, and the hypocellular fibrous pattern [14].

Although glucocorticoids therapy plays an essential role in early and benign lesions, historically, the IMT was often treated with surgical excision of the tumor, which is considered the gold standard in clinical practice [12]. Some authors strongly recommended a biopsy and intraoperative rapid pathological assessment for patients with only one kidney, bilateral masses, or kidney insufficiency because of the advantages of eliminating malignant tumors and preventing needless removal [2,8,12,14,15]. Moreover, the accurate diagnosis of IMT is usually only made after nephrectomy. Therefore, these approaches are still disputed. Kidney function is increasingly being saved thanks to the widespread use of partial nephrectomy [3,12].

Targeted treatment and chemotherapy are also adjuvants. ALK-targeted medicines like crizotinib are effective and give surgical alternatives for patients with metastatic or unresectable ALK-positive IMT [15]. Toxicity and resistance in tumor cells can reduce therapy efficacy. When ALK inhibition is not an option for ALK-negative tumors, the nonsteroidal anti-inflammatory drugs (NSAIDs) have been helpful [16]. It is also postulated that NSAIDs may shrink tumors and perhaps cure cancer [16]. Corticosteroids are also effective in young children with bilateral renal-infiltrating IMT. Moreover, radiotherapy has no role in renal IMT [13].

Renal IMT has historically shown favorable outcomes, evident from a study conducted by Kapusta et al., who found that there is no recurrence during 17 years of follow-up [17]. IMT outside the kidney has been associated with local recurrence and distant metastasis. Recurrence risk factors include positive surgical margins and abnormalities in DNA aneuploidy and the expression of the p53 gene [17]. However, some reports mention a possible association of IMT with renal cell carcinoma (RCC), so a long-term follow-up is recommended even if the disease has benign characteristics [10] (Table 1).

**Table 1:** Clinical characteristics of pediatric inflammatory myofibroblastic tumor of the kidney M: male, F: female, Rt: right, Lt: left, NA: not available, UPJ: ureteropelvic junction.

Case	Reference	Age (year)/gender	Location	Size (cm)	Clinical symptoms	Treatment	Follow-up (months)
1	Tarhan et al. [18]	10/F	Rt middle	NA	Fever, headache	Nephrectomy	18
2	Dogan et al. [4]	3/M	Rt upper	6	Fever, lower back pain	Nephrectomy	6
3	Ho et al. [19]	3/F	Lt UPJ	8	Abdominal pain, fever	Conservative surgery	9
4	Vujanić et al. [20]	8/M	Rt lower	6	Painless gross hematuria	Nephrectomy	36
5	Boo et al. [1]	9/F	Lt upper	5.5	Abdominal pain, weight loss	Nephrectomy and lymph node dissection	6
6	Present Case	9 (months)/F	Rt lower	4.7	Gross hematuria	Nephrectomy and lymph node dissection	Follow-up every 3 months for 2-3 years

## Conclusions

A rare neoplasm, renal IMT, has an unknown cause and manifests itself in a variety of ways clinically. Despite its rarity, it is critical to be aware of this entity to avoid misdiagnosis when a renal tumor, possible RCC, is suspected. In the current mainstream of treatment, surgery plays a key role, while glucocorticoids and NSAIDs in ALK-negative IMT play a pivotal role. Despite the benign nature in most cases, long-term follow-up is highly recommended due to possible hidden association with RCC.
